# Liquid nitrogen-cryopreserved tarsal plate and palpebral conjunctival transplantation for the repair of tarsal defects

**DOI:** 10.3892/etm.2013.1004

**Published:** 2013-03-12

**Authors:** YANG LIU, LIN-NONG WANG, LI-XUN CHEN

**Affiliations:** Department of Ophthalmology, Nanjing First Hospital Affiliated to Nanjing Medical University, Nanjing, Jiangsu 210006, P.R. China

**Keywords:** cryopreservation, donor tarsal plate and palpebral conjunctiva, reparation, tarsus defect

## Abstract

The objectives of this study were to compare the efficiency of three methods for repairing tarsal defects (glycerine-preserved, alcohol-preserved and cryopreserved tarsal plate and palpebral conjunctival transplantation) based on histopathological changes and apoptosis, and to evaluate the clinical effects of repairing tarsal defects by liquid nitrogen-cryopreserved tarsal plate and palpebral conjunctival transplantation. Forty-eight rabbit eyes with tarsal defects were equally divided into three groups and transplanted with tarsal plates and palpebral conjunctivae under the following conditions: Group 1, liquid nitrogen cryopreservation; Group 2, glycerine preservation; and Group 3, alcohol preservation. Histopathological changes in the transplants were observed by light microscopy 1 week, 1 month and 3 months postoperatively. Apoptosis was assessed by terminal deoxynucleotidyl transferase dUTP nick end labeling (TUNEL) staining. Clinically, transplantations of liquid nitrogen-cryopreserved tarsal plates and palpebral conjunctivae were performed in 30 eyes (29 cases) to repair tarsal defects caused by the excision of neoplasms. The postoperative inflammatory reaction and number of apoptotic cells in Group 1 were lower compared with those in Groups 2 and 3. Clinically, of the 30 eyes operated on, 14 were cured, 15 improved and 1 failed between 6 and 90 months of follow-up. Liquid nitrogen-cryopreserved tarsal plate and palpebral conjunctival transplantation is an easy, feasible and convenient procedure. Its effects are fairly favorable, with only a small rejection rate postoperatively. Therefore, it is an ideal method for repairing tarsal defects.

## Introduction

Eyelids are the protective barrier of eyeballs against any trauma. They not only protect the eyes from light and dust, but also moisturize and cleanse the cornea. The repair of tarsal defects is crucial in terms of both function and aesthetic quality. Transplantations of glycerine-preserved and liquid nitrogen-cryopreserved tarsal plates for the repair of tarsal defects have been demonstrated to yield certain clinical effects (1–3). However, studies on combined tarsal plate and palpebral conjunctival transplantation for such repair have not been reported to date. We performed an experimental study on tarsal plate and palpebral conjunctival transplantation for repairing tarsal defects in rabbits using glycerine preservation, alcohol preservation and liquid nitrogen cryopreservation between May 2006 and July 2007. We compared the groups on their clinical effects, histopathological rejection and apoptotic cells postoperatively. We also completed 30 surgeries for tarsal defects in patients caused by the excision of eyelid neoplasms using cryopreservation between March 2001 and October 2009 and obtained fairly favorable clinical outcomes.

## Materials and methods

### Animals

Twenty-four healthy Chinese white rabbits (weight, 2–3 kg) were sacrificed by air embolism. Forty-eight transplants were made from both the superior tarsal plates and the palpebral conjunctivae, and they were divided equally into three groups (with 16 implants per group): Group 1, liquid nitrogen cryopreservation; Group 2, glycerine preservation; and Group 3, alcohol preservation. All transplants were preserved for 6 months. The study was approved by the ethics committee of Nanjing First Hospital Affiliated to Nanjing Medical University, Nanjing, China. Written informed consent was obtained from the patient.

### Liquid nitrogen cryopreservation

Sixteen transplants were stored in a moist room after sterilization, placed in a 4°C thermostatic water tank and frozen for 20 min. Two concentrations of dimethyl sulfoxide solution (5 and 7.5%) were used with 20% albumin as a solvent. The transplants were placed in the two solutions, frozen for 10 min and then kept in a liquid nitrogen container at −196°C. When required, the transplants were taken out of the liquid nitrogen container and then immersed in a 40°C thermostatic water bath. Approximately 120 sec later, when the cryoprotective solutions had finished thawing, the samples were taken out using sterile tweezers and eventually immersed in saline. The samples were ready for use after soaking for 10 min.

### Glycerine preservation

Sixteen transplants were placed in a pure sterile glycerine bottle and refrigerated at 4°C. Twenty-four hours later, they were moved into another pure glycerine bottle and kept under sealed preserving conditions in a 4°C refrigerator. They were washed with saline before use and then immersed in gentamicin solution in the ratio of 1:3000. The transplants were ready for use after 15–20 min of revival.

### Alcohol preservation

Sixteen transplants were immersed in 75% alcohol. Three days later, they were moved to 95% alcohol. Within a tight container, they were refrigerated at 4°C and then washed with saline before use. They were immersed in gentamicin solution in the ratio of 1:3000. The transplants were ready for use after 15–20 min of revival.

### Animal models

Twenty-four healthy Chinese white rabbits weighing between 2 and 3 kg were equally divided into three groups (with 8 rabbits per group). We reconstructed 16 pairs of superior tarsi by tarsal plate and palpebral conjunctival transplantation in Groups 1–3. Pentobarbital sodium was injected into the vein of each rabbit at 30–35 mg/kg. After general anesthesia, tarsal defects were excised by more than half of the length of the upper eyelid palpebral margin. The transplants were then trimmed into the right size and shape. Tarsal and palpebral conjunctival stumps were closed by interrupted sutures with 6-0 absorbed thread. The subjects received topical application of tobramycin eye ointment for the next 2 weeks. Based on daily observation of the transplants, two animals in each group were sacrificed 1 week, 1 month and 3 months postoperatively; one animal in each group was sacrificed ∼4 months postoperatively; and three animals died without specific cause, one of the three were from Group 2, the others were from Group 3.

### Histological observations

Collected specimens were perfused with 10% neutral formalin and then cut into paraffin-embedded sections. All slices were stained with hematoxylin and eosin. Responses, including inflammatory infiltration, transplant tissue necrosis, connective tissue hyperplasia and glandular cells, were observed by light microscopy. We marked the paraffin-embedded sections at the 3′-end hydroxyl caused by apoptotic cell nuclear DNA cleavage. Apoptosis was observed under a fluoroscope. Positive nuclei were detected by double-blind observation. Using 10 randomly selected cells from each specimen, we read the number of apoptotic cells under high-power fields of vision and calculated the average value.

### Statistical analysis

Statistical analysis was performed with SPSS 11.0. Data were analyzed using the paired t-test. P<0.05 was considered to indicate a statistically significant result.

### Clinical observations

Twenty-nine patients (30 eyes; age range, 23–85 years) were admitted to our institution for tarsal defects caused by the excision of eyelid neoplasms between March 2001 and October 2009. One patient presented with right superior eyelid neoplasm recurrence after excision. This patient had undergone four surgeries on the right eye, including two excisions of superior eyelid neoplasms, allogeneic tarsal and palpebral conjunctival transplantation by cryopreservation and reconstruction of the superior eyelid in sequence. Of the 29 patients, 11 were male and 18 were female. Eleven patients had meibomian gland carcinoma, 9 had basal cell carcinoma, 6 had squamous cell carcinoma, 2 had conjunctival carcinoma *in situ* and 1 had sebaceous nevus. After neoplasm excision, 3 patients developed palpebral marginal defects less than one-third of the length of the eyelid, 11 developed palpebral marginal defects from one-third to half in length and 15 developed palpebral marginal defects more than half in length. The course of disease ranged between 1 month and 20 years.

### Preparation, preservation and thawing of allo-tarsal plates and palpebral conjunctivae

Palpebral conjunctivae from both living bodies and corpses (allo-tarsal plates and palpebral conjunctivae could not be separated) were obtained from 18-to 60-year-old donors who were selected according to EBAA standards. Within 6 h of the donors’ death, we prepared palpebral conjunctivae using the aseptic technique. The palpebral conjunctivae were stored in a moist chamber, refrigerated at 4°C and frozen for 20 min. Albumin (20%) was used as a solvent and mixed with gradually increasing concentrations of dimethyl sulfoxide solution (5 and 7.5%). The palpebral conjunctivae were then immersed in the two solutions and the mixtures were frozen for 10 min. A lower-temperature procedure was used to maintain the specimens at −80°C. Finally, specimens were stored in a −196°C liquid nitrogen container for 3 months to 5 years. When they were ready for use, the palpebral conjunctivae were taken out from the liquid nitrogen container, immersed in a 40°C constant water bath and swung slightly. When the frozen cryoprotective solutions had finished thawing, that is, only a thin layer of ice appeared around the palpebral conjunctivae, they were transferred to saline using sterile tweezers. The samples were ready for use after soaking for 10 min (4).

### Surgery

Eyelid subcutaneous tissues were collected and fornices with tissue infiltration were injected with anesthesia (2% lidocaine and 0.75% bupivacaine). Neoplasms were excised beyond 4–5 mm of their exterior. Rapidly frozen slices were subjected to pathological examination. If the tissue of a slice margin was considered normal, palpebral conjunctival transplantation was initiated. The tarsal palpebral conjunctivae, along with the eyelashes, were trimmed into the size and shape that were required. They were then sutured with the original tarsal stump or internal and external palpebral periorbital ligaments. If the case involved a superior tarsal defect, we kept the levator palpebrae superioris fixed on the upper eyelid margin. Palpebral conjunctivae were closed with an interrupted suture. Transferring or sliding the musculocutaneous flap can reconstruct skin defects. The correct tension was applied to avoid entropion and ectropion of the eyelids postoperatively. Dressings were postoperatively changed once to twice a day and pressed for 5 days. Local and general anti-biotics were used. Xibrom was applied to local sites. Stitches were removed 2 weeks postsurgery.

### Evaluation criteria of therapeutic effects

Cure refers to the morphological and functional recovery of the eyelids. The length and height differences of the palpebral fissure should both be <2 mm compared with normal eyes. Eyelids should be able to close well, and entropion and ectropion should not be observed. Improvement refers to a morphologically and functionally improved state of the eyelids. Compared with normal eyes, the palpebral fissure length difference should be no less than 2 mm, and its height difference should be no less than 2 mm. Eyelids should be able to close well, and entropion and ectropion should not be detected. A slight incision on the tarsal margin would be present. Invalidity refers to cases in which the eyelids do not exhibit any morphological and functional improvement, and cases in which implants separated.

## Results

### Implants

All implants from the three experimental groups exhibited responses including hyperemia and edema, among others, 1 day postoperatively. These responses reached a peak in the following 7–10 days but gradually disappeared. Among the three groups, the implants in Group 1 demonstrated milder responses. During the observation period, all tarsal plates and palpebral conjunctivae survived, and the surface of the palpebral conjunctivae was smooth.

### Morphological observations

**Figure 1:**
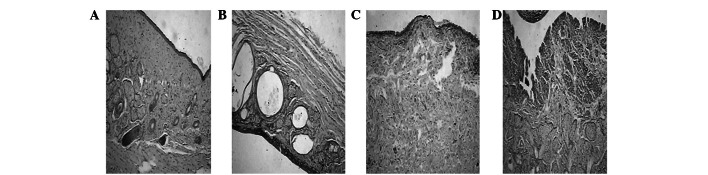
(A) In the glycerine-preserved group, the inflammation response decreased. The image depicts fiber connective tissue hyperplasia (×40), 3 months after transplantation. (B) In the alcohol-preserved group, the inflammation response was less steadily maintained. The image depicts connective tissue hyperplasia and extension to the sebaceous glands (×40), after controlling it for 3 months. (C) In the fluid nitrogen-cryopreserved group, the inflammation response kept less steady compared with the alcohol-preserved group. There was less collagen connective tissue hyperplasia compared with the two other groups (×40), after transplantation in the group. (D) In the fluid nitrogen-cryopreserved group, with the same conditions as in (C), a number of glands remained in the implants (×40), under local observation.

Three groups of stained specimens were observed by microscopy. During the first week, inflammatory infiltration, necrosis and gland necrosis were detected among them. Inflammatory infiltration disappeared within the first month. Collagen fiber and connective tissue hyperplasia were eventually observed. Three months later, inflammatory cells completely disappeared, but collagen fiber hyperplasia emerged. Milder inflammatory infiltration and less necrosis were observed in Group 1 in the early stage compared with the other two groups, but a number of gland cells did not survive. Group 1 also exhibited milder connective hyperplasia during the late period (Fig. 1).

### Terminal deoxynucleotidyl transferase dUTP nick end labeling (TUNEL) assay

**Figure 2:**
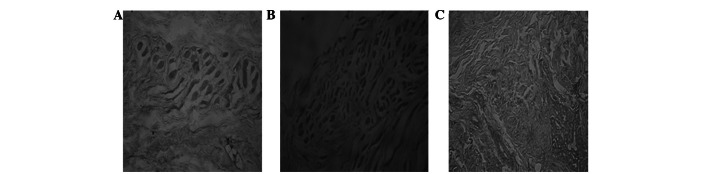
(A) Glycerine-preserved group, 3 months after transplantation (×40). (B) Alcohol-preserved group, 3 months after transplantation (×40). (C) Fluid nitrogen cryopreserved group, 3 months after transplantation (×40). Apoptosis was observed after transplantation in all three groups. The number of apoptotic cells in the fluid nitrogen cryopreserved group was lower than that of the other groups in sight.

Paraffin-embedded sections were specifically marked at the 3′-end hydroxyl caused by apoptotic cell nuclear DNA cleavage using TUNEL to detect DNA. Cells that had brown and yellow granules in the nucleus were considered positive (i.e., apoptotic cells). Apoptosis of all the transplants of tarsal plates and palpebral conjunctivae was observed in the three groups 1 week, 1 month and 3 months (Fig. 2) postoperatively. However, during the same period, the number of apoptotic cells in Group 1 was markedly lower compared with the numbers in Groups 2 and 3. Statistically significant (P<0.05) and insignificant (P>0.05) differences were noted (Table I).

### Clinical observations

**Figure 3:**
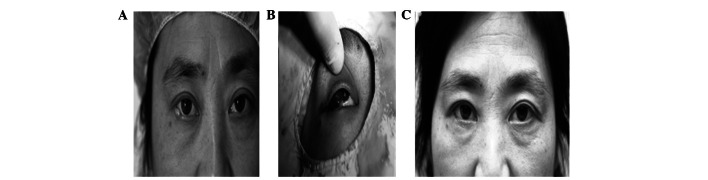
(A) Right lower eyelid neoplasm before surgery; (B) Tarsal defect, half palpebral margin in length, caused by completely excising the neoplasm during the surgery. Sutured tarsal palpebral conjunctiva in apposition to the defect. (C) With good appearance in 3rd month postoperatively, the palpebral fissure length difference compared with the healthy one was <2 mm and the height difference was <2 mm. Eyelids were able to close well. No entropion or ectropion were observed. The transplant of tarsal palpebral conjunctiva survived, and therefore, it was considered cured.

**Figure 4:**
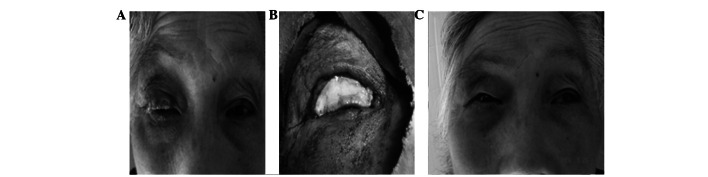
(A) Right upper neoplasm before surgery. (B) Tarsal defect, full tarsal margin in length, caused by completely excising neoplasm during the surgery. The trimmed tarsal plate and palpebral conjunctiva were inserted into the defect. (C) With good appearance in the 3rd month postoperatively, the palpebral fissure length difference compared with the healthy one was >2 mm and the height difference was >2 mm. Eyelids could close well. No entropion or ectropion were observed. Although light notching remained, the transplant of tarsal plate and palpebral conjunctiva survived, thus, it was improved.

Patients were followed for 6 to 90 months postoperatively (53.3 months on average) and none presented with recurrence. In 15 eyes, mild notching remained and light conjunctiva became shallow, whereas the rest of the eyelids recovered morphologically and functionally. Furthermore, the eyelids were able to close well, and postoperative entropion and ectropion were not observed. Transplantation failed in only one patient (1 eye) due to several reasons: First, the incisal edge residue from the initial surgery had not been completely excised. Next, when the tumor that expanded again was excised, we did not replace the tarsal plate and palpebral conjunctival transplant, as a result of which the original transplant separated in the following half month of the second surgery. Overall, 14 eyes were cured and 15 eyes improved in this group. The eyes of 3 patients in whom palpebral marginal defects measured less than one-third in length were cured. Of 11 cases of palpebral marginal defects measuring one-third to half in length, 8 eyes were cured and 3 improved (Fig. 3). Finally, of 15 cases of palpebral marginal defects measuring more than half in length, 3 eyes were cured and 12 improved (Fig. 4).

## Discussion

Procedures to repair tarsal defects can be selected and designed based on several factors, including patient age, the characteristics of the eyelids, the size and depth of the defect and different relationships with eyelid margins. Either sliding or repair of the transposition flap can be selected for cases with larger defects of the anterior eyelid (5). The first choice would be to use an interior chin myocutaneous flap (6). For significantly larger defects, thickness skin grafting may be adopted (7). The primary methods of tarsal repair include free graft transplantation (8), sliding, indexable palpebral conjunctival flap (9) and use of various tarsus substitutes, such as composite transplants of the nasal septal chondromucosal cartilage (10), allogeneic sclera (11), auricular cartilage (12), hard palatal mucosa (13), allogeneic dura mater (14) and tarsal plate (8), among others. Repair of palpebral conjunctival defects can be performed by sliding conjunctival fornices or autologous lip mucosae (5).

The tarsus is an integral component of eyelid tissue. Internal and external canthal ligaments and tarsi play vital roles in maintaining the appearance and stability of eyelids. Therefore, repairing the posterior laminar palpebral conjunctiva during the repair of eyelid defects warrants further investigation.

Either composite transplants of the nasal septal chondromucosa or ear cartilage tissue can easily lead to many sequelae, for instance, stiff eyelids, poor activity and thickening eyelids, among others. Thus, our surgery was limited to repairing only the lower eyelid defect. Most significantly with regard to the transplant of nasal mucosa, inappropriate use of feather and chamfer edges can cause necrosis of the nasal mucosa postoperatively. Drawing materials from the costicartilage is more difficult. Moreover, its curve and thickness differ from those of the tarsus, which is why such materials have to be further processed. The postoperative appearance associated with these materials is not good, and palpebral conjunctivae require transplanting. Although the thickness and curve of ear cartilage tissue are close to those of the tarsus, palpebral conjunctivae also require transplantation. However, inappropriate performance of the surgery may lead to auricle atrophy and distortion. Tarsal defects can also be repaired via transplantation of hard palatal mucosa, which works better; however, this method requires another wound.

Some advanced methods using the Mustarde technique (to repair the upper eyelid using the lower eyelid full-thickness rotation flap), Cutler-Beard technique (to repair the lower eyelid using the lower eyelid full-thickness sliding flap) and Hughes technique (for upper tarsus palpebral conjunctival sliding instead of the inner part of the lower eyelid defect), among others, may be considered. These technologies are made from the best materials, but their use is largely restricted and can damage healthy eyelids, rendering them unideal.

Autogenic materials outside the eyelid are not only mainly composed of collagenous fibers but also approximate the eyelid’s structure, making them convenient for transplants. In fact, they are not as hard as tarsi. Owing to lack of sustenance, they would not reach satisfactory stability with the repair of lower eyelid defects. Furthermore, due to absorption in various degrees after the transplantation of allogeneic sclera, palpebral conjunctivae would require transplanting.

Tarsal plate and palpebral conjunctival transplantation is widely reported in the literature. We have repaired palpebral conjunctival defects by mostly performing conjunctival metastasis repair. For the repair of larger tarsal defects, we combined allogeneic tarsal plate and palpebral conjunctival transplantation, instead of using sliding autologous conjunctival fornix or autologous lip mucosa transplantation, and obtained nearly optimal outcomes. Only one case failed in our observation. The eyes of all the other patients were cured or improved. With a smooth palpebral conjunctival surface, symblepharon and tarsal collapse did not occur after the reconstruction of the eyelid, thereby reducing damage to the autologous conjunctival fornix. As to the repair of tarsal defects, the combined allogeneic tarsal plate and palpebral conjunctival transplantation could take the place of tarsal plate transplantation, sliding autologous conjunctival fornix transplantation and autologous lip mucosa transplantation.

The advantages of a combined tarsal plate and palpebral conjunctival transplantation procedure are as follows: i) the tarsal plate is similar to the autogenous tarsus in terms of thickness, curve and toughness, and the eyelid margin closely approximates with the physiological state. This procedure allows the right size of tarsus to be used, even the full tarsus, according to the exact site of the eyelid defect. It yields fairly satisfactory results; ii) it makes it easy to fix the levator palpebrae superioris and avoids ptosis of the eyelid and limited movement; iii) it is associated with lower immunogenicity; iv) under this procedure, tarsal plates and palpebral conjunctivae may also avoid stimulation on cartilage that lacks a smooth surface, thereby reducing the risk of corneal injury; v) it allows for convenient selection of materials with simple solution and preserving conditions (2).

The advantages of tarsal plate and palpebral conjunctival transplantation in practical applications are as follows: i) this procedure does not require complex decoration on the tarsal plate as the combined effect of the method is extremely effective. On account of the toughness of the tarsus and its flexible shape, the risk for postoperative deformation is lower under this procedure; ii) this procedure could avoid or reduce trauma in other parts of the body.

In the animal experiments, all tarsal plate and palpebral conjunctival transplants were preserved under three conditions. During the first week, the three groups responded differently to the transplantation with inflammatory infiltration, necrosis or gland necrosis. Within the first month, inflammatory infiltration had disappeared. Collagen fiber and connective tissue hyperplasias then appeared. In Group 3, the alcohol-preserved group, which was affected by physical and chemical factors, proteins of allo-tissue structure became inactive and some surface antigenic determinants even disappeared. However, some determinants of antigen molecules emerged. Denatured protein represented decreasing antigenicity, but new antigenic specificity followed. Therefore, in Group 3, the substitutions of allo-tarsus had no activity but exhibited antigenicity (11). The regular dry agent glycerine was inactive during the preservation of tarsal plates and palpebral conjunctivae. In fact, glycerine served as an antiseptic preservative (15).

Compared with glycerine and alcohol preservation, liquid nitrogen cryopreservation, in which cell energy metabolism is completely inhibited, is a novel method. Cells subjected to cryopreservation become so dormant that they are preserved for a long time. After thawing under proper conditions, the cells are restored to metabolize, exerting their biological functions. Thus, this type of preservation is active (4). Furthermore, much research has shown that cryopreservation reduces the immunogenicity of tissue to a certain degree (16). In the present study, milder inflammatory infiltration and less necrosis were observed in Group 1, the cryopreserved group, in the early stage compared with Groups 2 and 3, but a number of gland cells did not survive. A milder case of connective hyperplasia was also observed in Group 1 during the late period.

Apoptosis after organ transplantation is correlated with a series of processes, such as graft preservation, ischemia, reperfusion injury, immunological rejection reaction and immunological tolerance (6,17–20). Apoptosis and necrosis are the characteristic morphological changes in allograft rejection. The levels of apoptosis are positively correlated with the degree of rejection (21). By contrast, apoptosis eliminates the cellular immunity of the host so as to induce immune tolerance (22). Studies have confirmed that cytotoxic T lymphocyte (CTL)-mediated cytotoxicity is the major cause of immunological rejection. CTL-mediated apoptosis primarily results from the Fas/FasL system, whereas the killing effects of CTLs on target cells are exerted by apoptotic pathways. The coexistence of apoptosis and necrosis in target cells is attributable to CTLs (3). These data suggest that CTLs, which are involved in immunological rejection, lead to transplant injury.

In the present study, all specimens from the three groups exhibited apoptotic cells under postoperative TEM and SEM observations. However, the number of apoptotic cells in Group 1 was lower compared with the numbers in Groups 2 and 3 within the same period. These results showed that cryopreservation has significant advantages over the traditional glycerine and alcohol preservation methods in terms of preservation and postoperative rejection. Cryopreservation was performed only in an eye bank, making it extremely safe and convenient to obtain, preserve and assign tarsal plates and palpebral conjunctivae, thus providing us with broader space for our basic work.

Alloantigens, which were detected in the tarsal plates and palpebral conjunctivae, may have caused the postoperative rejection documented in this study. To repair conjunctival defects, we mainly performed conjunctival metastasis repair. If the defect was larger, we performed transplantation of human autologous mucosa. An allogeneic tarsus is the same as a broad tissue receptor. Its fewer blood vessels and inactivity characterize it as a weak antigen.

Antibodies in the tissue could be tolerated postoperatively and do not cause evident immune responses, which makes healing satisfactory (23,24). However, we repaired tarsal defects of experimental animals in this group by combined allo-tarsal palpebral conjunctival transplantation. We did not find any animal model of graft failure caused only by local and general rejection. Allogeneic tissue grafts inevitably cause immune responses, but research has shown that slight immune responses should be beneficial for the healing of implants and organisms (25). Stimulation of implants, lymphocytes and eosinophils trigger numerous reactions, including the formation of granulation tissue and mother cells of fibroblasts. As a result, local nutrition is strengthened and metabolism is improved. In the process of transplantation, antibodies of body fluid also develop. Antibodies are capable of facilitating transplant damage; however, they are also able to protect transplants against cell responses (i.e., immunoenhancement). Non-cytotoxic antibodies may contribute to the survival of transplants. After having been transplanted into a receptor for a long time, a graft gradually adapts to the receptor. When allo-transplant immunogenicity decreases, on the contrary, the receptor would gradually increase its immunological tolerance and no longer exhibit an immune response to the transplant.

It is in this light that we did not observe any failure of transplantation caused by rejection among the rabbits after tarsal plate and palpebral conjunctival transplantation in the present study. Neovascularization increasingly developed into the tarsal plate and palpebral conjunctival margin after transplantation according to our postoperative clinical observations. Within the first 2 months postsurgery, amyotrophy of parts of the tarsal tissue appeared and the tissue became thinner. However, the figure of the eyelid had already been reconstructed at that time, and it had no effect on the outcomes of surgery. This study has demonstrated that liquid nitrogen-cryopreserved tarsal plate and palpebral conjunctival transplantation is an ideal method for repairing tarsal defects. As this procedure remains largely unexplored, the pathological changes and immune responses post-transplantation warrant further investigation.

## Figures and Tables

**Table I:** Comparison of the number of apoptotic cells in the rabbits’ tarsal plate and palpebral conjunctival transplantation.

Group	n	1st week	1st month	≥3rd month
Cryopreserved group (A)	16	10.69±2.31	17.63±2.00	16.19±2.63
Glycerine-preserved group (B)	16	18.50±3.38	24.69±3.19	26.00±3.19
Alcohol-preserved group (C)	16	19.00±2.19	26.81±2.69	26.63±2.50
P-value				
A/B		<0.01	0.03	0.03
A/C		<0.01	0.03	0.02
B/C		0.14	0.26	0.46

Cryopreservation refers to the −196°C fluid nitrogen preservation.
